# Exploring Left Atrial Appendage Thrombi in Large Vessel Occlusion Stroke by Cardiac CT: Thrombus Features, LAA Characteristics and the Impact of Direct Oral Anticoagulation

**DOI:** 10.3390/neurolint17080127

**Published:** 2025-08-11

**Authors:** Karim Mostafa, Sarah Krutmann, Cosima Wünsche, Naomi Larsen, Alexander Seiler, Hatim Seoudy, Domagoj Schunk, Olav Jansen, Patrick Langguth

**Affiliations:** 1 Department of Radiology and Neuroradiology, University Medical Center Schleswig-Holstein, Campus Kiel, 24103 Kiel, Germany; cosima.wuensche02@uksh.de (C.W.); naomi.larsen@uksh.de (N.L.); olav.jansen@uksh.de (O.J.); patrick.langguth@uksh.de (P.L.); 2 Department of Neurology, University Medical Center Schleswig-Holstein, Campus Kiel, 24103 Kiel, Germany; sarah.krutmann@uksh.de (S.K.); alexander.seiler@uksh.de (A.S.); 3 Department of Internal Medicine III, Cardiology, Angiology and Critical Care, University Medical Center Schleswig-Holstein, 24103 Kiel, Germany; hatim.seoudy@uksh.de; 4 Interdisciplinary Emergency Department, University Medical Center Schleswig-Holstein, Campus Kiel, 24103 Kiel, Germany; domagoj.schunk@uksh.de; 5 Clinician Scientist Programme, Faculty of Medicine, University of Kiel, 24103 Kiel, Germany

**Keywords:** computed tomography angiography, cardiac imaging techniques, stroke, anticoagulant therapy, stroke/diagnostic imaging, cardiology, neurology

## Abstract

**Background:** Large vessel occlusion (LVO) strokes account for a significant proportion of ischemic strokes and are often cardioembolic in origin, particularly following atrial fibrillation (AF) with thrombus formation in the left atrial appendage (LAA). Although direct oral anticoagulation (DOAC) therapy reduces stroke risk in AF, anatomical and flow-related factors may still allow thrombi to form and persist, revealing the limitations of anticoagulation in high-risk patients. Examining structural and hemodynamic factors contributing to thrombus persistence is essential for optimizing patient management. **Methods:** We retrospectively analyzed 169 AF patients with LVO stroke who underwent cardiac CT (cCT) during acute stroke assessment. Patients were categorized based on the presence or absence of persistent LAA thrombi and further stratified by DOAC status. LAA volume, blood stasis and left ventricular (LV) diameter were measured. Thrombi were assessed using Hounsfield Unit (HU) analysis to evaluate potential differences in thrombus composition. Logistic regression analysis was performed to identify independent predictors of thrombus persistence with adjustment for DOAC therapy. **Results:** Persistent LAA thrombi were identified in 23 patients (13.6%). Patients with thrombi had significantly higher rates of stasis (*p* = 0.004), larger left ventricular diameters (*p* = 0.0019) and higher LAA volumes (*p* = 0.004). When adjusted for DOAC therapy, larger LAA volume (OR 1.05, *p* = 0.011), presence of LAA stasis (OR 6.14, *p* = 0.013) and increased LV diameter (OR 1.06, *p* = 0.006) were independent predictors of thrombus persistence. Thrombus size and HU values did not differ significantly between DOAC and non-DOAC groups. Notably, 30.4% of patients with persistent thrombi were on adequate DOAC therapy. **Conclusions:** LAA volume, stasis and LV enlargement predict thrombus persistence in the LAA of AF patients with LVO stroke, even under adequate DOAC therapy. These findings highlight the potential need for alternative antithrombotic strategies, including interventional LAA occlusion, and warrant further investigation into individualized stroke prevention in high-risk AF populations.

## 1. Introduction

Cerebral ischemia due to large vessel occlusion (LVO) accounts for approximately 30% of all ischemic strokes and is associated with a more severe clinical presentation and a substantial increase in morbidity and mortality compared to strokes of other causes [1,2]. LVOs are typically caused by either cardioembolism or atherosclerosis of the large arteries [3]. The most common etiology of cardioembolism is atrial fibrillation (AF) with subsequent intracardial thrombus formation [4,5,6]. In AF, effective stroke prevention is usually achieved through oral anticoagulation treatment (OAC) [7]. In cardioembolic stroke attributed to AF, OAC with direct oral anticoagulants (DOAC) can reduce the rate of stroke recurrence by up to 65% and mortality by up to 25% [8,9,10]. However, despite optimized anticoagulation therapy, both first-ever and recurrent strokes during OAC treatment continue to remain of high clinical relevance [11,12,13,14,15]. The exact underlying pathophysiological mechanisms for this phenomenon are still not fully understood. Various factors have been proposed including competing stroke etiologies, nutritional interactions, advanced atrial disease or lack of adherence to OAC treatment [8,16,17,18].

**Figure 1:**
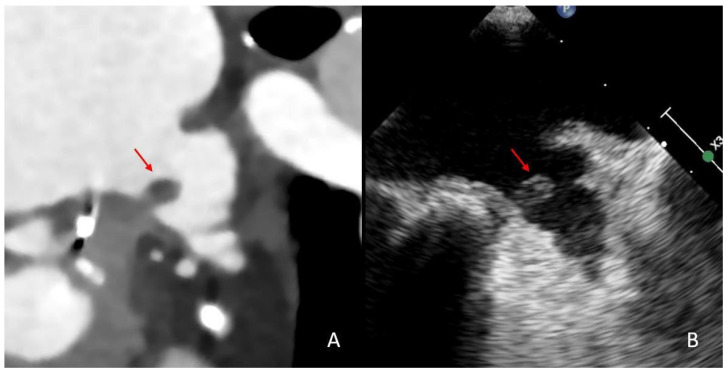
Comparison of thrombus imaging in the LAA on cardiac CT and transesophageal echocardiography in the same patient. This patient presented with an acute stroke with occlusion of the basilar artery. Upon cCT imaging, a thrombus can be depicted proximally in the LAA (red arrow, image (**A**)). The thrombus was still detectable on TEE three days after the stroke event (red arrow, image (**B**)).

In recent years, cardiac CT (cCT), alongside transesophageal (TEE) and transthoracic echocardiography (TTE), has evolved into a rapid, high-yield and versatile imaging technique for the comprehensive assessment of cardioembolic causes in acute stroke (Figure 1) [19,20,21]. In this context, a special diagnostic focus is placed on the left atrial appendage (LAA) which is recognized as an area of high vulnerability to thrombus formation, particularly in patients with AF. In regard to the LAA morphology and its functional properties, cCT has been demonstrated to provide an accurate assessment of thrombi in this location. The diagnostic precision of cCT has been further improved by using modern CT technologies such as spectral imaging which allow for an advanced differentiation between thrombi and LAA stasis [19,22,23,24].

In light of this background, the objective of this study was to investigate the LAA structure and LAA thrombi in the context of LVO and AF using cCT. We analyzed the morphological characteristics of the LAA in patients with and without LAA thrombi, focusing on LAA configurations, LAA volumes and the presence of LAA blood stasis as well as thrombus characteristics. Furthermore, in patients with LAA thrombi, we assessed the impact of an established or discontinued OAC treatment on thrombus and LAA properties.

## 2. Methods

This study was approved by the local ethics committee and was conducted in accordance with the STROBE guidelines and the ethical standards of the declaration of Helsinki of 1964 and its later amendments (File no. D 524/48, date of approval: 29 July 2024).

### 2.1. Patient Selection and Data Collection

**Scheme 1:**
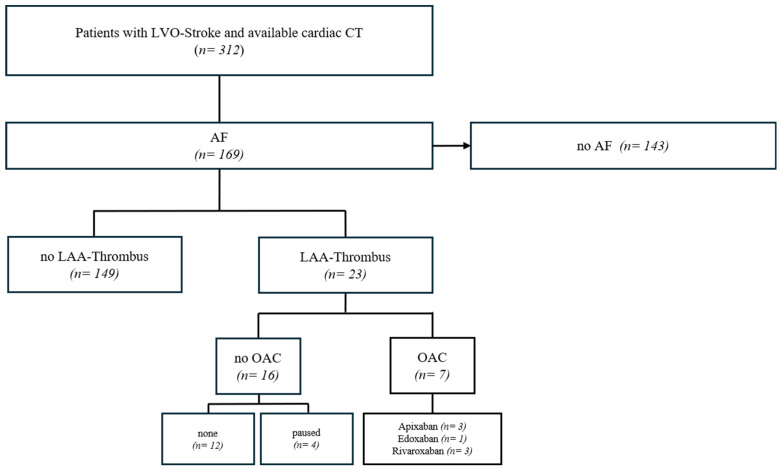
Patient selection flowchart. LVO—large vessel occlusion; AF—atrial fibrillation; LAA—left atrial appendage; OAC—oral anticoagulation.

From our database of 313 cCT examinations in the acute phase of an LVO stroke, we retrospectively identified all patients with newly diagnosed or previously known AF. The database includes cases from January 2018 to December 2024. All strokes were classified as cardioembolic by interdisciplinary consensus. The AF patients were divided into two groups: (1) patients without thrombi in the LAA and (2) patients with thrombi in the LAA. Secondly, we further subdivided all patients with thrombi in the LAA into two groups: (a) patients who were receiving OAC treatment at the time of stroke and (b) patients without OAC treatment. In addition, further clinical baseline data on cardiovascular risk factors and comorbidities such as smoking, diabetes, dyslipidemia, heart failure and previous strokes or heart attacks were obtained (Scheme 1).

### 2.2. Assessment of Anticoagulation Status

Types and dosages of OAC therapy as well as adherence were evaluated based on thorough assessment of the medical records and information obtained from the patients, their relatives or caregivers. This primary assessment was conducted in the acute setting, as this information was crucial for potential intravenous thrombolysis therapy. Retrospectively, we further validated anticoagulation adequacy considering type, dosages and appropriateness for patient-specific factors. Blood level tests were reviewed given their availability. Patients were classified as having adequate OAC therapy when type, dosage and schedule were correct with appropriate adjustments for age, weight and renal function following accepted guidelines [25,26]. Inappropriate dosages and uncertain adherences were classified as inadequate OAC therapy.

### 2.3. Cardiac CT Imaging Acquisition

The cCT imaging was performed on two different CT systems (IQon and iCT; Philips Healthcare, Best, The Netherlands) with ECG gating in our tertiary stroke center. These images were acquired immediately after multimodal stroke imaging including CT angiography and perfusion study of the brain. The following scan parameters were used for the cCT: 64 × 0.625 mm collimation, 0.27 s gantry rotation time, 100 kV or 120 kV tube voltage and 375 mA tube current.

After completion of brain imaging with a total of 80 mL of contrast agent (Imeron, Bracco, Milan, Italy), an additional 45 mL bolus of contrast agent was used for cCT, resulting in a total contrast agent dose for the entire examination of 125 mL per patient. No additional medications were administered for cCT acquisition.

### 2.4. Imaging Analysis

The cCT images were analyzed by two reviewers with over 10 and over 3 years of experience in cardiac imaging, respectively. Intracardiac thrombi and stasis in the LAA were assessed, and discrepant results were resolved by consensus reading. In addition, LAA length, depth and height were measured in multiplanar reconstructions, and LAA volume was approximated in ml [(length × depth × height in mm)/1000]. The configurations of the LAA were divided into four shape categories: chicken wing, cauliflower, cactus and windsock [27]. Furthermore, Hounsfield units (HU) were measured at multiple positions, including the LAA, LAA stasis, the left atrium and the ascending aorta.

**Figure 2:**
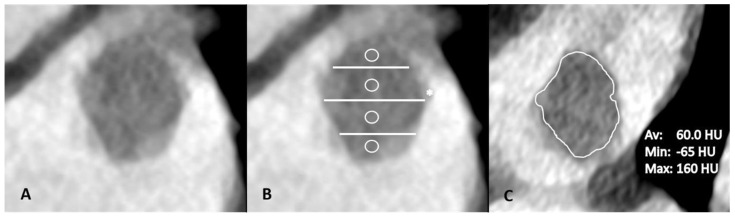
Thrombus HU measurements. This LAA thrombus is seen to have its maximum diameter in craniocaudal orientation (**A**). The thrombus is then divided into four parts, each marked with a circle (**B**). A free-hand ROI in axial orientation is then drawn in all four parts of the thrombus in axial orientation to assess the minimum, average and maximum HU values of the slice, yielding a total of 12 measurements per thrombus ((**B**), star; (**C**)).

The thrombi were measured in three dimensions and divided into four sections based on their longest dimension. Multiplanar reconstructions were generated using external software (Philips Intellispace, Best, The Netherlands) and thrombi were marked using a free-hand ROI. This allowed the minimum, average and maximum HU values to be determined in each of the four sections, resulting in a total of 12 measurement points per thrombus (Figure 2). Thrombus volume was calculated in ml [(length × height × width in mm)/1000], and the percentage of LAA volume accounted for by thrombus volume was also determined.

Finally, left ventricular dilatation was measured in multiplanar reconstruction in three-chamber view according to the American Society of Echocardiography chamber measurement guideline [28].

### 2.5. Statistical Analysis

**Figure 3:**
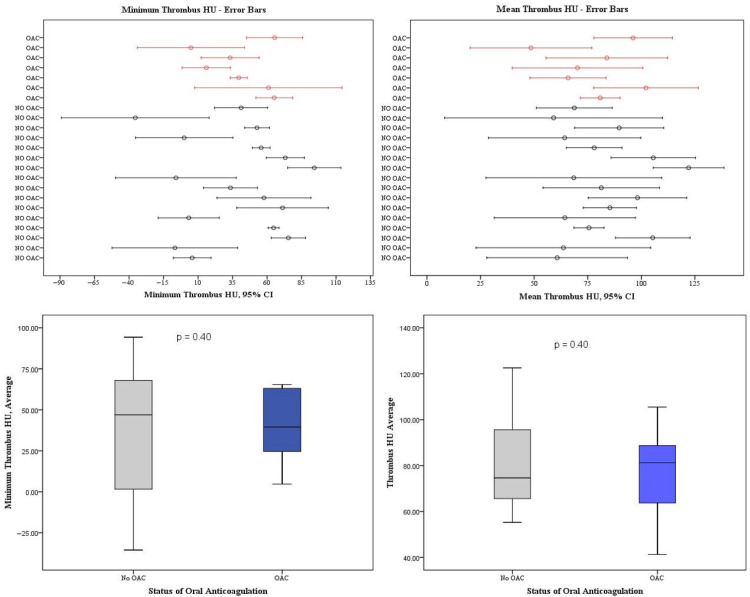
**Error bar diagrams and boxplots for minimum and mean thrombus HU stratified by oral anticoagulation status.** The top row represents error bar plots depicting mean and standard deviation of minimum thrombus HU (**upper left**) and mean thrombus HU (**upper right**) for every patient. Patients with DOAC treatment are shown in red, while those without DOAC are black. The bottom row displays corresponding boxplots for minimum HU (**lower left**) and mean HU (**lower right**). These visualizations demonstrate that thrombus HU values do not significantly differ between patients with and without DOAC treatment, reinforcing the notion that DOAC status does not influence thrombus configuration.

The analysis was performed with the statistical software IBM SPSS 25 (Armonk, NY, USA). The mean minimum and the average thrombus HU values per patient were presented as error bar charts (Figure 3). Thrombus HU values, thrombus volume, presence of stasis, percentage of thrombus in the LAA, HU aorta/HU stasis ratio, LAA configurations and LV diameter measurements were compared between the two subgroups using a T-test or chi-square test as indicated. Logistic regression analysis was performed to determine factors influencing thrombus persistence with correction for OAC treatment. Interaction terms for OAC treatment were calculated. Selected results were additionally presented in boxplots. Significance was set at *p* < 0.05.

## 3. Results

### 3.1. Patient Characteristics

Of the 312 patients with a cCT in the acute phase of an LVO stroke, we identified a total of 169 patients with known or new-onset AF (n = 119 female, mean age 80.4 ± 10.6 years, Table 1). Overall, 42 patients received adequate OAC therapy, whereby DOAC agents were used in all cases. The main clinical and laboratory findings for the patient collective are summarized in Table 1.

### 3.2. Subgroup Analysis: Patients with LAA Persistent Thrombi vs. Patients Without Persistent LAA Thrombi

**Figure 4:**
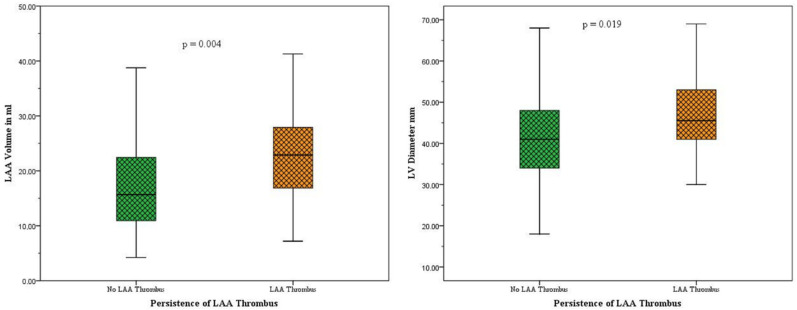
**Boxplots depicting LAA volumes and LV diameters in presence or absence of persistent LAA thrombi.** The left boxplots illustrate LAA volume distribution in patients with persistent LAA thrombi (orange) and without persistent LAA thrombi (green), while the boxplots on the left represent LV diameter distributions. The LAA volumes of patients with thrombi were seen to be significantly higher compared to patients without thrombi (LAA volumes: no thrombus group: 17.7 ± 9.5 mL; thrombus group: 23.7 ± 10.3 mL, *p* = 0.004). Furthermore, significantly higher LV diameters were seen in patients with persistent thrombi in the LAA (LV diameter: no thrombus group: 41.4 ± 10.1 mm; thrombus group: 48.2 ± 11.8 mm, *p* = 0.019).

In the subgroup of patients with a thrombus in the LAA (n = 23), we found a significantly higher volume and a significantly higher prevalence of LAA stasis compared to patients without a thrombus (LAA volumes: no thrombus group: 17.7 ± 9.5 mL, thrombus group: 23.7 ± 10.3 mL, *p* = 0.007; LAA stasis: no thrombus group: 39 of 148, in thrombus group: 21 of 23; *p* = 0.001; Figure 4). In addition, the mean left ventricular diameter was significantly larger in the thrombus group than in the group without thrombus (LV diameter: no thrombus group: 41.4 ± 10.1 mm, thrombus group: 48.2 ± 11.8 mm, *p* = 0.003; Figure 4). We found no significant differences between LAA configuration and HU ratio of the aorta and LAA stasis. Similarly, there were no significant differences between the two groups in terms of clinical and laboratory findings (Table 1).

### 3.3. Logistic Regression Analysis

Unadjusted logistic regression analysis confirmed the LAA volume, LAA stasis and LV diameter as factors significantly associated with thrombus persistence (LAA volume: OR 1.05, 95% CI: 1.01–1.10, *p* = 0.010; LAA stasis OR 6.73, 95% CI: 1.52–29.78, *p* = 0.012; LV diameter OR 1.06, 95% CI: 1.02–1.11, *p* = 0.005 (Table 2)).

After adjustment for adequate DOAC therapy status all factors remained significant predictors for thrombus persistence (LAA volume: OR 1.05, 95%CI: 1.01–1.11, *p* = 0.011; LAA stasis OR 6.14, 95% CI: 1.49–29.55, *p* = 0.013; LV diameter OR 1.06, 95% CI: 1.02–1.11, *p* = 0.006 (Table 2)). Interaction terms were included in the regression model, whereby no statistical significance was detected, indicating no relationship to adequate OAC treatment (LV diameter × DOAC: *p* = 0.143; LAA volume × DOAC: *p* = 0.639; LAA stasis × DOAC: *p* = 0.224).

### 3.4. Subgroup Analysis: LAA Thrombi Despite OAC Treatment vs. LAA Thrombi Without OAC Treatment

**Figure 5:**
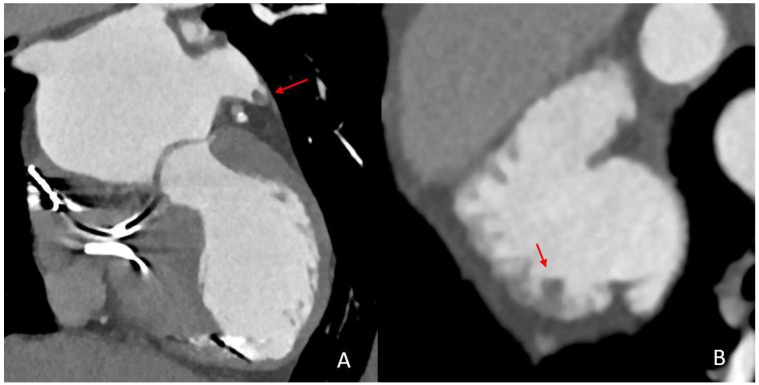
**Thrombus in the cauliflower-shaped LAA.** In this patient with LVO, a persistent thrombus was depicted on cCT in the framework of stroke etiology assessment (red arrow, image (**A**)). The LAA exhibits a distinct cauliflower shape with multiple small indentations, which can be delineated in multiplanar reconstructions on the CT imaging (red arrow, image (**B**)).

We identified a total of 23 patients (13.2%) of the 169 patients with new onset or known AF with a thrombus in the LAA on the cCT images (17 women, mean age 79.0 ± 9.5 years, Supplementary Material, Table S1). The majority (n = 16) were either not on DOAC or had appropriately discontinued OAC treatment, while 7 patients had a thrombus despite appropriate DOAC therapy. Comparison between DOAC-treated vs. non-DOAC-treated patients in thrombus volume (*p* = 0.34), minimum (*p* = 0.40) or average HU (*p* = 0.40) of the thrombus, percentage of thrombus in the LAA (*p* = 0.50) or HU ratio of aorta and LAA stasis (*p* = 0.69) showed no significant differences (Supplementary Table S1, Figure 3). Maximum thrombus HU values were not further evaluated due to artifacts of the surrounding contrast agent in the LAA and potential attenuation effects of the thrombus, rendering the HU measurements too high for a reasonable analysis (Mean 122.8 HU, range 88.8–149.5 HU). Furthermore, no significant differences in LAA configurations were detectable between the groups (Figure 5). The groups also did not differ significantly in terms of clinical or laboratory findings (Supplementary Table S1).

## 4. Discussion

The main findings of this study are as follows:

(1) AF Patients with persistent LAA thrombi after cardioembolic LVO stroke exhibited significantly larger LAA volumes, a higher prevalence of LAA stasis and increased left ventricular diameters compared to patients with AF without LAA thrombi (Figure 4, Table 1).
(2) LAA volume, LAA stasis and LV diameter are independent predictors of thrombus persistence in the LAA irrespective of an adequate DOAC therapy (Table 2).
(3) Persistent LAA thrombi in AF patients after LVO stroke under adequate OAC treatment do not differ in thrombus volume and HU values from those in AF patients without DOAC therapy (Figure 3, Table S1).

### 4.1. Assessment of LAA Thrombi Under DOAC

The findings of the present study demonstrate that LAA thrombi in patients with DOAC therapy do not vary in terms of size or minimum or average HU values when assessed with conventional CT images. As shown in an in vitro study by Kirchof et al., CT can be used to differentiate between different types of thrombi [29]. Considering the mean average HU of 79.2 and the minimum HU of 37.7 in our cohort, these thrombi are probably of mixed platelet and fibrinous composition, which is in line with the study by Kirchof et al. However, we also saw negative HU values for parts of some thrombi, suggesting fatty thrombus components. Phagocytosis of platelets with accumulation of fat during thrombus degeneration is a described phenomenon, which may potentially underlie this finding [30]. However, we did not observe a significant difference in lipid-containing thrombus components in the DOAC and non-DOAC groups. This challenges the hypothesis that failure of DOAC in terms of the prevention of thrombus formation is associated with higher lipid content in thrombi, suggesting that alternative mechanisms may underlie DOAC failure in these cases.

Furthermore, we did not identify specific LAA configurations associated with an increased risk of thrombus persistence. In particular, the previously hypothesized increased thrombogenic potential of cauliflower-shaped LAAs and the lower rate of thrombus formation for chicken wing-shaped LAAs could not be confirmed in our study (Figure 5) [31,32].

### 4.2. Predictors of LAA Thrombus Persistence in AF Patients with Cardioembolic LVO Stroke

The physiological contraction of the LAA during the cardiac cycle ensures a continuous blood flow and helps prevent thrombus formation. However, this mechanism is substantially impaired in AF, leading to the pathomorphological correlate of LAA stasis [31]. In our study, we found significantly higher LAA volumes in AF patients with thrombi compared to those without thrombi (Table 1) and we demonstrated that LAA volume was independently associated with thrombus persistence, even in patients receiving DOAC treatment. Increased LAA volume is a known predictor of embolic stroke, which has been shown in several studies and is reflected in our cohort [33,34]. In addition, larger LAA volume has been linked to recurrent and refractory AF, underlining its association to atrial cardiopathy and thrombogenesis [35,36].

A common imaging finding in AF is LAA stasis, which can manifest as a filling defect on cCT or as spontaneous echo contrast (SEC) on TEE [22]. In our cohort of patients with LVO stroke and AF, we observed LAA stasis in 64.9% of cases, including a rate of 91.3% in the subgroup of patients with AF and LAA thrombus. This was significantly higher than in the overall AF group and it was independently associated with thrombus persistence, irrespective of DOAC treatment (Table 2). Data in the literature regarding LAA stasis in patients with AF vary widely due to the heterogeneity of study designs. Teunissen et al. reported a stasis rate of 4.3% in 477 patients undergoing elective cardioversion for symptomatic AF, although the exact rate of previous stroke is not mentioned [37]. Notably, this rate of LAA stasis is considerably lower than in our study. This discrepancy can be attributed to the specific properties of the study population of patients with AF and LVO stroke. This hypothesis is further supported by the findings of Lui et al., who reported a significantly higher rate of LAA filling defects in patients with previous cardioembolic stroke compared to patients without stroke in 152 patients with AF (44.4% vs. 18%) [38]. Nevertheless, this number remains considerably lower than in our cohort. It should be noted that our study population was significantly older and included only patients with cCT imaging in the acute phase of LVO stroke compared to both mentioned studies, which may offer another explanation for the increased detection rate of LAA stasis in our study.

Finally, beyond parameters specific to the LAA, patients with AF and thrombi in the LAA were found to have larger left ventricular diameters compared to patients without LAA thrombi and it was also found to be an independent predictor of thrombus persistence, irrespective of DOAC treatment. The association between heart failure, left ventricular hypertrophy, reduced ejection fraction, AF and LAA thrombogenicity has been well studied [35,36,37].

However, contradictory findings exist in regard to the LV diameter. In a multicenter study of 1066 patients with AF, moderate to severe systolic dysfunction was shown to increase the risk of stroke, but the LV diameter was not identified as an independent risk factor [38]. In another study of 768 patients prior to AF ablation, reduced left ventricular ejection fraction was significantly associated with LAA thrombus, but not with changes in LV diameter [39]. However, it needs to be noted that in both studies the stroke rate was low at 7.3% and 6.8%, respectively. In 2020, Song et al. identified LV enlargement on echocardiography as a predictor of stroke in a case-control study of 233 patients with non-valvular AF and thrombus or SEC in the LAA [40]. In their study, 17.2% of the high-risk patients, defined as presence of thrombi or SEC in the LAA, had suffered a previous stroke. Their reported mean diameter of 50.1 mm in the high-risk group is in good agreement with our measurements of 48.2 mm. However, the main difference to their study is that our population consists only of high-risk patients with LAA thrombi. Nonetheless, our results are in good agreement with this study.

### 4.3. Summary and Clinical Implications

In summary, our data suggest that in patients with AF and LVO stroke, LAA volume, LV diameter and presence of LAA stasis are predictors of thrombus persistence in the LAA, even if patients receive adequate oral anticoagulation therapy. These findings highlight potential limitations of DOAC therapy alone in preventing thrombus formation and persistence. Consequently, it may be speculated that alternative antithrombotic management strategies, including interventional LAA occlusion, may be a viable option to further reduce the risk of cardioembolic events in this high-risk patient cohort [41,42].

### 4.4. Limitations and Future Directions

This study has some limitations. Firstly, it is based exclusively on patients with LVO strokes. Secondly, apart from the retrospective design, it is also important to highlight that the determination of thrombus composition on contrast-enhanced cCT using HU values cannot be fully differentiated, as thrombi may be partially contrast-enhancing. Thirdly, while LAA stasis was seen to be a significant predictor of thrombus persistence, the width of the confidence interval suggests heterogeneity. The status of DOAC treatment was determined based on medical history and self-report without laboratory confirmation, which may introduce a degree of misclassification. Additionally, given the method of CT imaging acquisition, we were unable to consistently perform left ventricular diameter measurements exactly at end-diastole and LAA volume was approximated using a simplified dimensional formula, which does not account for morphological variability of the LAA. In the present study, systemic inflammatory biomarkers, such as neutrophil-to-lymphocyte ratio (NLR) and platelet-to-lymphocyte ratio (PLR), were not specifically assessed. In light of growing evidence on the role of chronic inflammation influencing both thrombotic and bleeding risk, a comprehensive assessment incorporating systemic inflammatory biomarkers, especially PLR and NLR, alongside anatomical and functional imaging parameters may enhance future risk stratification strategies in atrial fibrillation and aid in balancing the need for and risk of a more aggressive antithrombotic therapy [43,44,45,46]. As for the topic of atrial fibrillation and cardioembolic stroke, elevated NLR has been shown to be an independent risk factor for stroke of cardioembolic origin in patients with atrial fibrillation and it has furthermore been identified as an independent risk factor for poor prognosis in after stroke [43,44]. In light of our research, these results suggest a potential interplay of morphological and systemic factors that remains to be fully elucidated. This provides an interesting gateway for future research.

## 5. Conclusions

In patients with large vessel occlusion stroke under atrial fibrillation, a higher LAA volume, an enlarged LV diameter and presence of LAA stasis are predictors of thrombus persistence in the LAA, independent of an adequate oral anticoagulation therapy. Alternative antithrombotic management options may be needed in such cases.

Persistent thrombi in the LAA of patients with large vessel occlusion stroke under atrial fibrillation with adequate oral anticoagulation do not differ in size or composition from persistent thrombi in patients without anticoagulation in CT imaging.

## Figures and Tables

**Table 1:** Baseline Characteristics and Imaging Findings in Patients with and without Persistent LAA Thrombus.

	Overall (n = 169)	No LAA Thrombus (n = 146)	LAA Thrombus (n = 23)	*p*-Value
Sex	119 f, 50 m	102 f, 41 m	17 f, 6 m	
Age, years	80.4 ± 10.6	80.6 ± 10.8	79.3 ± 9.5	0.50
**Imaging Findings**				
LAA Chicken Wing, n	81 (47.9%)	72 (49.3%)	9 (39.1%)	0.39
LAA Cauliflower, n	37 (21.9%)	30 (20.5%)	7 (30.4%)	0.27
LAA Cactus, n	15 (8.9%)	14 (9.6%)	1 (4.3%)	0.42
LAA Windsock, n	38 (22.5%)	32 (21.9%)	6 (26.1%)	0.63
LAA Volume (ml), avg.	18.5 ± 9.8 mL	17.7 ± 9.5 mL	23.7 ± 10.3 mL	0.004 **
LAA Stasis, n	111 (65.7%)	90 (61.2%)	21 (91.3%)	0.004 **
Mean LV Diameter (mm), avg.	42.3 ± 10.6 mm	41.5 ± 10.1 mm	48.5 ± 12.1 mm	0.019 **
HU Ratio Aorta/Stasis, avg.	2.9 ± 1.7	2.7 ± 1.6	3.3 ± 1.2	0.12
**Clinical and Laboratory Findings**				
History of Smoking, n	33 (19.5%)	29 (19.9%)	4 (17.4%)	0.80
Diabetes, n	30 (17.8%)	25 (17.1%)	5 (21.7%)	0.57
Dyslipidemia, n	38 (22.5%)	35 (24.0%)	3 (13.0%)	0.26
Hypertension, n	115 (68.0%)	101 (69.2%)	14 (60.9%)	0.49
History of Stroke, n	34 (20.1%)	27 (18.5%)	7 (30.4%)	0.17
History of heart failure, n	18 (10.7%)	16 (11.0%)	2 (8.7%)	0.33

** Indicates significant results.

**Table 2:** Independent Factors Associated with Thrombus Persistence in Patients with Atrial Fibrillation.

Adjustment	Factors	Odds-Ratio	CI 95%	*p*-Value
**Unadjusted**	LV Diameter	**1.06**	**1.02–1.11**	0.005 **
	LAA Volume	**1.05**	**1.01–1.10**	0.010 *
	LAA Stasis	**6.73**	**1.52–29.78**	0.012 *
**Adjusted for DOAC**	LV Diameter	**1.06**	**1.02–1.11**	0.006 **
	LAA Volume	**1.05**	**1.01–1.11**	0.011 *
	LAA Stasis	**6.14**	**1.49–29.55**	0.013 *
**DOAC Interaction Effect**	LV Diameter	**---**	**---**	**0.143**
	LAA Volume	**---**	**---**	**0.639**
	LAA Stasis	**---**	**---**	**0.224**

* *p* < 0.05; ** *p* < 0.01.

## Data Availability

The datasets analyzed during the current study are available from the corresponding author on reasonable request.

## Supplementary Materials

The following supporting information can be downloaded at: https://www.mdpi.com/article/10.3390/neurolint17080127/s1, Table S1. Comparison of all patients with AF and thrombi.
