# Supplementary material for: Exploring Left Atrial Appendage Thrombi in Large Vessel Occlusion Stroke by Cardiac CT: Thrombus Features, LAA Characteristics and the Impact of Direct Oral Anticoagulation
Source: Neurol Int. 2025 Aug 11;17(8):127. doi: 10.3390/neurolint17080127 (PMC12388839; doi:10.3390/neurolint17080127)
Supplement: Supplementary file 1 [file neurolint-17-00127-s001.zip › neurolint-3761376-supplementary.pdf]

Exploring Left Atrial Appendage Thrombi in Large Vessel Occlusion Stroke by Cardiac CT: Thrombus Features, LAA Characteristics and the Impact of Anticoagulation

Supplementary Material

Supplementary Table S1. Comparison of all patients with AF and thrombi

	Overall (n=23)	No OAC (n = 16)	OAC (n = 7)	p
Gender		10f, 6m	7f, 0m	
Age	79.4 ± 9.5	79.6 ± 10.1	78.9 ± 8.6	0.86
**Imaging Findings**				
LAA Chicken Wing, n, %	9 (39.1%)	7 (43.8%)	2 (28.6%)	0.49
LAA Cauliflower, n, %	7 (30.4%)	4 (25.0%)	3 (42.9%)	0.39
LAA Cactus, n, %	1 (4.3%)	0	1 (14.3%)	0.12
LAA Windsock, n, %	6 (26.1%)	5 (31.3%)	1 (14.3%)	0.39
LAA Stasis, n, %	21 (91.3%)	15 (93.8%)	6 (85.7%)	0.53
HU Ratio Aorta/Stasis, avg.	3.3 ± 1.2	3.2 ± 1.2	3.5 ± 1.3	0.69
Thrombus Volume, avg.	1.8 ± 2.3 ml	1.8 ± 2.2 ml	1.8 ± 2.8 ml	0.34
Min. Thrombus HU, avg.	37.7 ± 33.9 HU	36.3 ± 37.9 HU	40.8 ± 24.5 HU	0.40
Avg. Thrombus HU, avg.	79.2 ± 19.6 HU	80.7 ± 19.1 HU	76.1 ± 21.7 HU	0.40
% Thrombus of LAA	4.3, IQR 13.9	3.8, IQR 17.4	4.3, IQR 4.3	0.50
Mean LV diameter	48.2 ± 11.8	49.5 ± 12.8	45.6 ± 9.8	0.47
**Clinical and Laboratory Findings**				
History of Smoking, n, %	4 (17.4%)	2 (12.5%)	2 (28.6%)	0.35
Diabetes, n, %	5 (21.7%)	2 (12.5%)	3 (42.9%)	0.10
Hypertension, n, %	14 (60.9%)	9 (56.3%)	5 (71.4%)	0.49
History of Stroke, n, %	7 (30.4%)	4 (25.0%)	3 (42.9%)	0.39
History of heart failure, n, %	2 (8.7%)	2 (12.5%)	0	0.33
**Atrial Fibrillation**				
New-onset, n	11 (47.8%)	10 (62.5%)	1 (6.3%)	0.04
Preexisting, n	12 (52.2%)	6 (37.5%)	6 (37.5%)	0.50
OAC discontinued >48h, n	3 (13.0%)	3 (18.8%)	0	
